# Treatment of canine cognitive dysfunction with novel butyrylcholinesterase inhibitor

**DOI:** 10.1038/s41598-021-97404-2

**Published:** 2021-09-13

**Authors:** Maja Zakošek Pipan, Sonja Prpar Mihevc, Malan Štrbenc, Urban Košak, Ilija German Ilić, Jurij Trontelj, Simon Žakelj, Stanislav Gobec, Darja Pavlin, Gregor Majdič

**Affiliations:** 1 Clinic for Reproduction and Large Animals, Veterinary Faculty, University of Ljubljana, Gerbičeva 60, Ljubljana, Slovenia; 2 Institute for Preclinical Sciences, Veterinary Faculty, University of Ljubljana, Gerbičeva 60, 1000 Ljubljana, Slovenia; 3 Faculty of Pharmacy, University of Ljubljana, Aškerčeva cesta 7, Ljubljana, Slovenia; 4 Clinic for Small Animals, Veterinary Faculty, University of Ljubljana, Gerbičeva 60, Ljubljana, Slovenia

**Keywords:** Neurology, Dementia, Neurodegeneration, Neurodegenerative diseases, Drug development, Alzheimer's disease

## Abstract

Canine cognitive dysfunction (CCD) is common in aged dogs and has many similarities with Alzheimer’s disease. Unfortunately, like Alzheimer’s disease, CCD cannot be cured. In the present study, we treated dogs with CCD with our newly developed and characterized butyrylcholinesterase inhibitor (BChEi). Seventeen dogs were randomized into two groups (treated with BChEi and untreated) and followed for 6 months at regular check-ups. The dogs’ cognitive status was determined by a Canine Dementia Scale (CADES) questionnaire and two cognitive tests. In dogs with moderate cognitive impairment, treatment caused significant improvement in the clinical rating of cognitive abilities and the performance-based tests of cognitive functioning when compared to the untreated group (*p* < 0.001). Dogs treated with BChEi showed markedly improved cognitive function with enhanced quality of life. No side effects were observed in the treated dogs with moderate cognitive impairment. According to the results of this preliminary study, there is an indication that novel BChEi may be a promising drug for the treatment of CCD in dogs and may be an interesting candidate for the treatment of Alzheimer's disease in humans. However, further clinical studies are needed to confirm this.

## Introduction

Neurodegenerative diseases are an increasing health problem. Alzheimer’s disease (AD) is one of the most severe neurodegenerative diseases, with the numbers of affected individuals rising exponentially worldwide^1^. The disease now affects nearly 50 million people, and the incidence is predicted to triple by 2050^1^. Similar illness in dogs, canine cognitive dysfunction (CCD) is estimated to affect more than 30 million dogs in the USA and over 15 million dogs in Europe^2^. CCD affects up to 60% of older dogs, mostly dogs older than 11 years^3^, and age is the most prominent risk factor for the development of this disease^4,5^.

CCD, also referred to as cognitive dysfunction syndrome (CDS), has many similarities with AD in humans^6^. Patients with AD or CCD show similar neuropathological changes affecting cerebral gyri (cerebral atrophy), cerebral angiopathy, and ventricular enlargement^7^. When these diseases progress, patients gradually lose their ability to communicate and show diminished cognitive abilities^8^. Clinically CCD includes several behavioral alterations such as disorientation, decreased social interaction, changes in sleep–wake pattern, elimination patterns, reduced locomotion, increased anxiety, and deficits in learning and memory^9^. The diagnosis of CCD is based on the recognition of behavioral signs and by excluding other medical causes that might mimic CCD or complicate its diagnosis. Like AD, CCD cannot be cured. Due to the high prevalence of age-related dementias in people and dogs, it is imperative to find a suitable medication for these devastating diseases.

One of the prominent signs of AD is cholinergic dysfunction^10^. Current treatment options for AD are only symptomatic, and three out of four available drugs are cholinesterase inhibitors^10^. Pharmacological interventions are beneficial as they temporarily improve cognition^11^, although they cannot stop the AD’s progression. Unfortunately, there have been more than 2000 failed AD clinical trials^12^, while more than 200 are currently ongoing. Some trialed drugs have been tested in dogs and often had similar effects reported for AD patients^13–16^.

Similarly to human AD, cholinergic hypofunction is present in dogs with CCD^13^, and this system is, therefore, an interesting target for the symptomatic treatment of CCD. Such treatments could work through the modulation of acetylcholinesterase (AChE) and butyrylcholinesterase (BChE), which are important enzymes of the cholinergic system. We have recently developed a range of selective nanomolar butyrylcholinesterase inhibitors (BChEi)^17,18^. One of the compounds has low cytotoxicity and crosses the blood–brain barrier in mice^17^. However, because the laboratory rodents do not develop spontaneous neurodegenerative diseases and human AD is only partially mimicked by the transgenic rodent models, dogs with CCD serve as a much better animal model for studying novel treatment options for AD. In the current study, we treated dogs with CCD with our newly developed and characterized BChEi. Dogs were administered BChEi for at least six months and showed marked improvements in their cognitive functions, assessed by the Canine Dementia Scale questionnaire (CADES score) and by two cognitive performance tests.

## Results

### Sample characteristics

Dogs included in the study were of different breeds, and all dogs were small to middle-sized. Breeds of dogs are listed in the supplemental material (Supplement 2—Table 2). Female dogs (64.7%) outnumbered male dogs (35.3%), but there was no statistically significant difference for sex between treated and untreated groups.

The mean age of dogs was 14.4 ± 1.3 years (range: 13–17 years). There was no statistically significant difference in age between treated (14.2 ± 1.3 years) and untreated (14.8 ± 1.3 years) groups. All females were spayed, and all males were intact.

### Disease severity and staging

Eleven dogs had moderate cognitive impairment, and 6 dogs had severe cognitive impairment at the start of the study. Seven dogs with moderate cognitive impairment and three dogs with severe cognitive impairment were included in the treatment group. We had to stop treatment in all dogs (3) with severe cognitive impairment since they all showed signs of gastrointestinal problems with vomiting and diarrhea after treatment. All dogs with moderate cognitive impairment continued the treatment without any noticeable adverse effects for 6 months.

**Figure 1:**
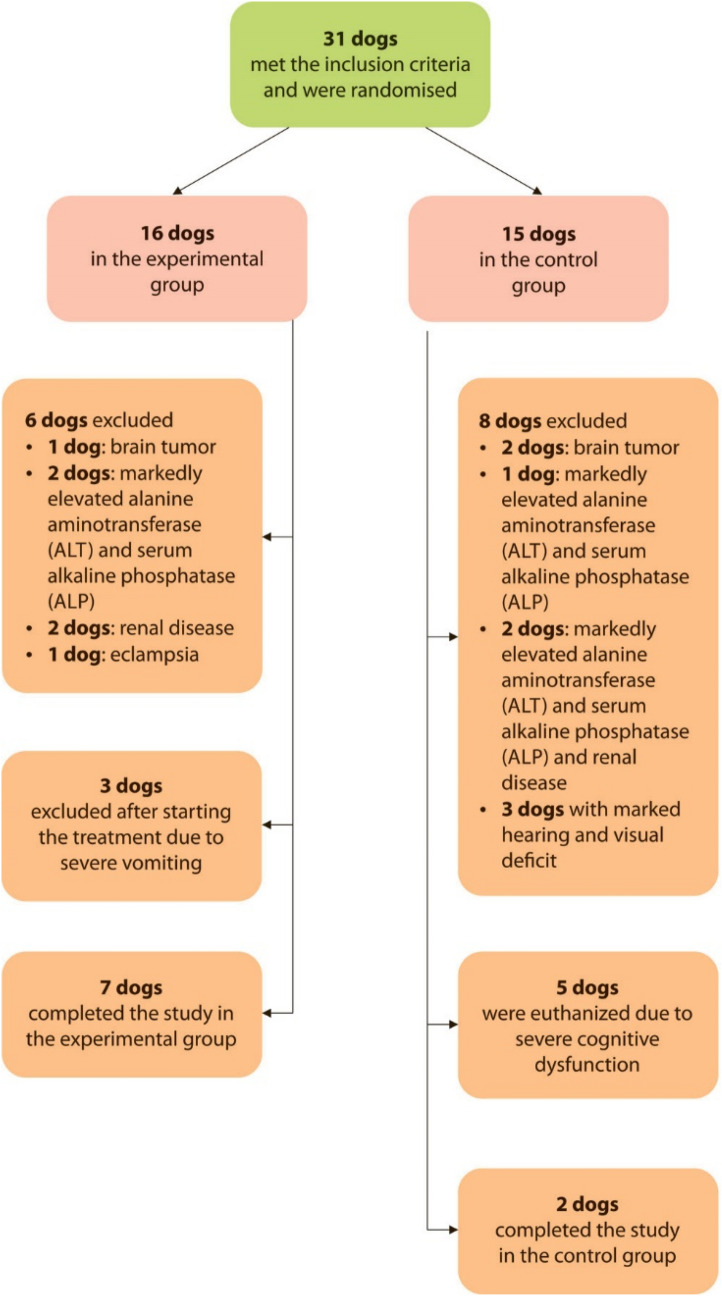
Flow-chart of patient recruitment.

There was no statistically significant difference in CADES scores between treated and untreated groups at the beginning of the study (Fig. 1). The number of affected domains in dogs is presented in Table 1.

**Table 1:** Dogs’ cognitive status based on CADES score and their affected domains at the beginning of the study.

Cognitive state	Affected cognitive domains			
Moderate cognitive impairment (all dogs)	Domain AB 4 (36.4%)	Domain BC 5 (45.5%)	Domain ABC 2 (18.2%)	/
Control group	2 (18.2%)	3 (27.3%)	2 (18.2%)	/
Experimental group	2 (18.2%)	2 (18.2%)	/	/
Severe cognitive impairment (all dogs)	Domain ABC 1 (16.7%)	Domain ACD 1 (16.7%)	Domain BCD 2 (33.3%)	Domain ABCD 2 (33.3%)
Control group	/	1 (16.7%)	1 (16.7%)	1 (16.7%)
Experimental group	1 (16.7%)	/	1 (16.7%)	1 (16.7%)

### CADES score

**Figure 2:**
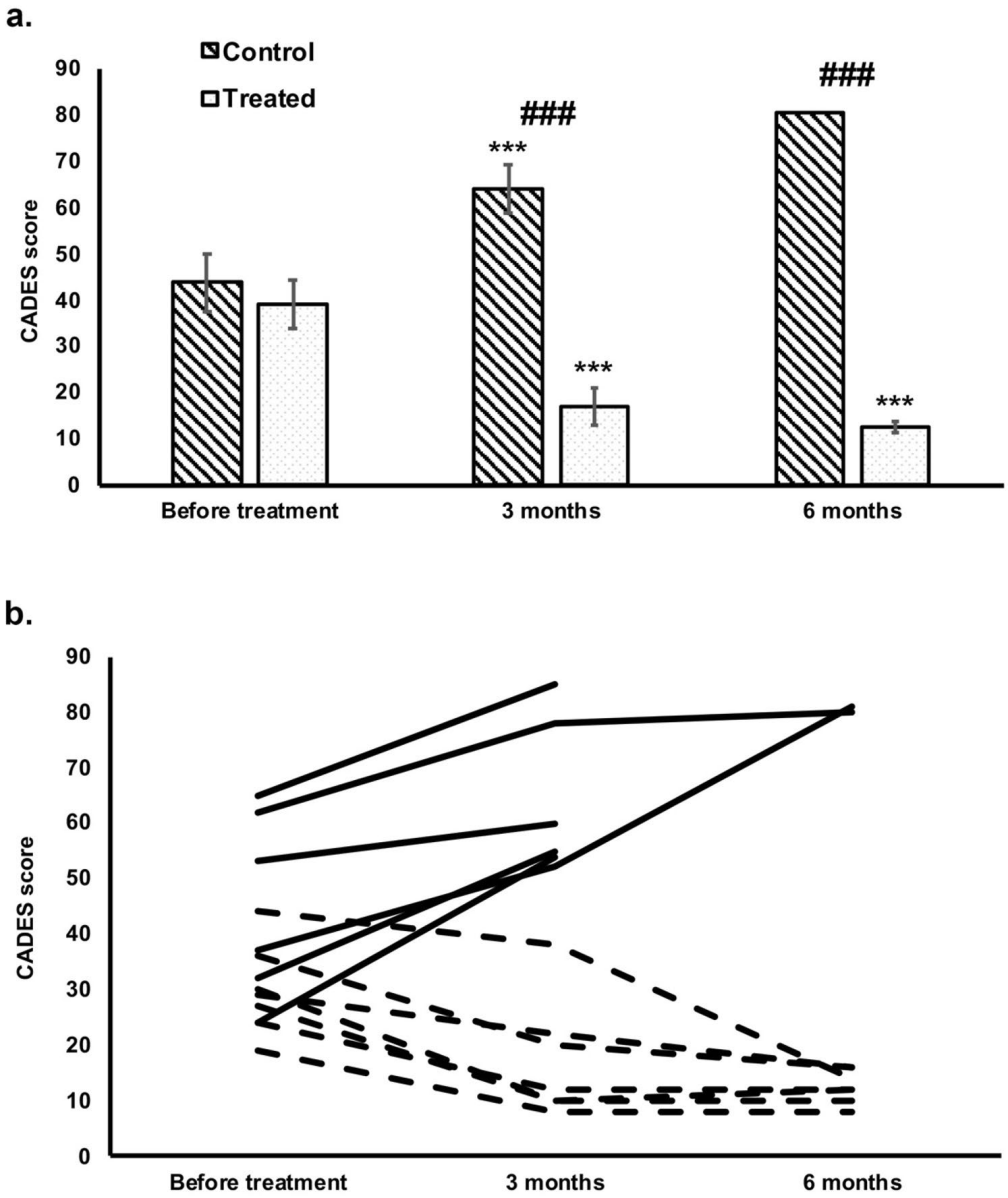
CADES score before and during the therapy in treated and control (untreated) dogs (**a**,**b**). At the beginning of the study, there was no difference in the CADES score between the treated and untreated groups. After three months, the CADES score was significantly higher in the untreated group (***, *p* < 0.001, **a**) and significantly lower in the treated group (*, *p* < 0.05, **a**). After six months, only two dogs in the untreated group remained alive (**b**), while all dogs were alive in the treated group (**b**) and showed significant improvement in CADES score in comparison to the score at the beginning of the study (***, *p* < 0.001, **a**). There was a significant difference between treated and untreated groups both at 3 and 6 months after the beginning of the treatment (###, *p* < 0.001, **a**). In panel **b**, broken lines represent treated dogs and unbroken lines untreated dogs.

There was a statistically significant decline in cognitive impairment in the untreated group after three months in all seven patients (*p* < 0.001; Fig. 2). Five out of seven dogs in the untreated group were euthanized due to severe cognitive and physical decline between 3rd and 5th month after the start of the trial. The other two dogs in the untreated group remained until the end of the study, but further decline in cognitive function was noted (Fig. 2).

In the treated group all dogs remained alive, and their CADES scores statistically significantly improved at 3 (*p* < 0.05) and 6 months (*p* < 0.001; Fig. 2) in comparison to the beginning of the study. The difference in CADES scores between untreated and treated groups was significantly different at 3 months and 6 months (*p* < 0.001; Fig. 2).

### Affected domains on CADES scale

To identify the cognitive impairment’s phenotypic variability, the number of dogs with impairment of selective domains was quantified. After three months, 42.8% of untreated dogs had extreme cognitive impairment with all domains (A, B, C, and D) affected. Severe cognitive impairment with at least three domains affected (A, C, and D) was observed in 57.2% of untreated dogs. The remaining two dogs had an extreme cognitive impairment according to CADES score with all four domains affected (A, B, C, and D) after 6 months.

Only 14.3% of dogs in the treated group showed severe cognitive impairment with three affected domains (A, C, and D) after three months. In others (85.7%), moderate cognitive impairment was noticed, with only two affected domains. After six months, all dogs showed moderate cognitive impairment with a low CADES score (Fig. 2). In all treated dogs, improved memory, cognitive functions, and learning abilities were noticed. The remaining symptoms owners observed were mostly reduced ability to perform previously learned tasks and to learn new tasks. Some dogs still had problems with house soiling, but less frequently. None of the dogs had problems during sleeping, and they did not show signs of anxiety. All owners of treated dogs reported a drastic improvement in the quality of life and dog-owner interaction.

### Problem-solving tests

**Figure 3:**
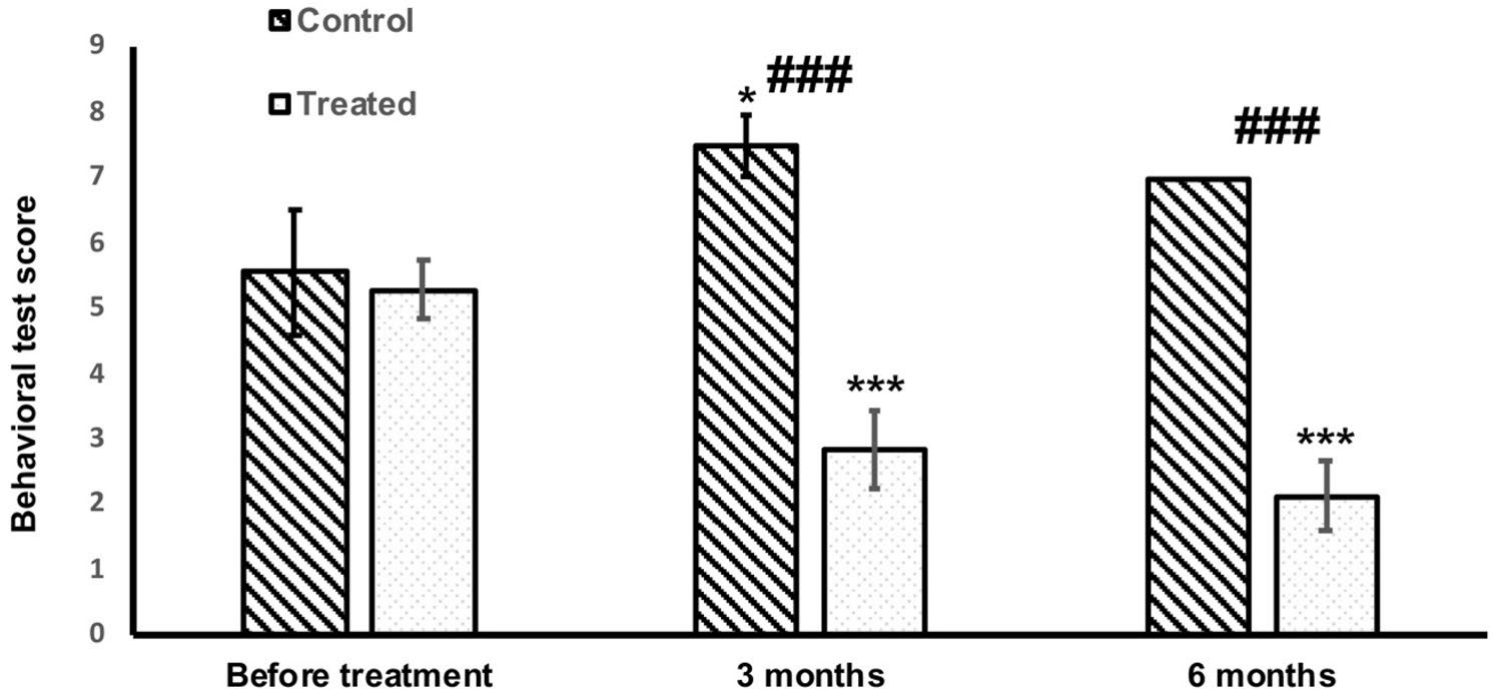
Problem-solving tests score before and during the treatment in treated and untreated groups. At the beginning of the study, there was no significant difference between treated and untreated dogs. In untreated dogs, the problem-solving task was significantly worse after 3 months (*, *p* < 0.05), while in the treated group, there was a significant improvement at both 3 and 6 months (***, *p* < 0.001). There was a statistically significant difference between treated and untreated groups at both 3 and 6 months (###, *p* < 0.001).

There was no difference in problem -solving tests at the beginning of the study between treated and untreated groups (Fig. 3). After three months, most untreated dogs (85.7%) did not show any attempt to search for the food (FST) and did not sniff the box where the food was hidden (PST). Only two dogs remained in the study at six months. In one of these dogs, the problem-solving score remained the same as at three months. In the other dog, a further decline was observed. However, an average score in the untreated group was slightly lower at 6 months since euthanized dogs were not included (Fig. 3).

In contrast, treated dogs could solve the tests better after three months and performed even better after six months. The difference between treated and untreated groups was statistically significant with *p* < 0.001 (Fig. 3).

## Discussion

This study shows that a newly developed BChEi could be used in treating cognitive decline in dogs. In dogs with moderate cognitive impairment, treatment was associated with the improved clinical rating of cognitive abilities. Furthermore, an improvement in the performance-based tests of cognitive functioning in dogs was observed.

To evaluate the severity of cognitive impairment and the effect of BChEi treatment on CCD signs, a questionnaire based on the CADES scale and two problem-solving tests were used. Rating scales are essential tools for CCD diagnosis and staging, but also for careful monitoring of the disease symptoms and evaluation of the treatment effects^19^. To reduce the subjective evaluation of dogs’ cognitive status based on the CADES questionnaire, all dogs included in the study were regularly checked by the participating veterinarian. Moreover, a baseline health check that included an assessment of all body systems and an extensive neurological examination (behavior and consciousness, cranial nerves, gait and posture, postural reactions, spinal reflexes) were performed to exclude other conditions that can mimic the symptoms of dementia. Consistent behavioral changes in spontaneous activity and the frequency of abnormal behavior were also reported and regularly checked by owners.

All dogs included in the study had moderate or severe cognitive impairment at the time of the enrollment. No dogs had mild cognitive impairment. It is well known that CCD is a severely under-diagnosed disease in aged dogs^5^. Since CCD is a complex disorder that involves many factors that impact the development of this disease, mild cognitive impairment is often mistaken by owners for normal aging in dogs^20^. In our study, owners observed signs of cognitive impairment in their dogs when a moderate cognitive impairment was already present. The most common symptoms were anxiousness, impairment in their social interactions, and sleep–wake cycles. This is in agreement with the previous study by Madari et al.^19^, who found that 70% of dogs with moderate cognitive impairment had impaired social activities and sleep–wake cycles. The same study revealed that 24% of dogs with mild cognitive impairment convert to moderate within the 6 months, but the study did not monitor the progression from moderate to severe. The quick progression of the disease was also observed in our study in untreated dogs. During the first three months, 28.7% of untreated dogs progressed from moderate to severe impairment. In the next three months, either the dogs were euthanized due to the severity of the disease or their cognitive functions further deteriorated. Likewise, after just three months, a decline in behavioral test solving abilities was noticed in the untreated group. With these findings, we confirmed previous observations on CCD dogs that even a mild cognitive impairment may present a strong predictor for the onset of full-blown CCD in older dogs^19^. However, when CCD in dogs was discovered and treated early in our study, a significant improvement in cognitive abilities was noticed. After six months, all treated dogs showed moderate cognitive impairment with statistically significantly lower CADES scores. They were also better at solving the FST and PTS after three months of treatment and even better after six months. The same was found in the original study using the FST and PST for the FST test, which found that younger dogs (< 9 years) were able to locate food faster and with more success than the older group (≥ 9 years) and dogs with severe CCD performed worse than those with mild CCD or their healthy counterparts. However, they found no differences in the ability to solve the PST related to the severity of CCD^21^. This was not the case in our study, where differences were found in relation to the severity of CCD.

All treated dogs stayed alive throughout the study, and owners were truly pleased, claiming that the dogs’ quality of life, and theirs, had greatly improved. Even though owner’s ratings of the dogs’ behavior may have been subjective, behavioral changes that were reported by owners included improvement in symptoms that are usually the most disruptive problems (sleeping during the night, social activities, interactions with other dogs, level of alertness) and are unlikely to be reported subjectively.

As presented here, the timing of the treatment seems to be of paramount importance. All dogs with severe cognitive impairment in our study presented with vomiting and nausea after the treatment initiation. These were likely the results of the muscarinic effects of the BChEi. However, where the new BChEi with selective reversible nanomolar BChE inhibition was given to dogs with moderate cognitive impairment, no side effects were observed throughout the study. This suggests that the novel inhibitor does not trigger muscarinic effects during the early stages of the disease. The same observation was also noticed in a study conducted on mice^17^, where no acute cholinergic adverse effects were seen even when mice were treated with a high dosage (100 mg/kg). Unfortunately, mice are not a perfect model because they do not spontaneously develop cognitive impairment^22^.

In humans with AD, a supportive co-regulating role of BChE in the brain cholinergic system was reported, and a negative role predicted elevated levels of BChE dysregulating brain AChE levels^23^. The basis for the adverse effects of approved drugs for treating patients with AD is the inhibition of cholinesterases in the basal ganglia, peripheral nervous system, and parasympathetic autonomic nervous system resulting in excess of acetylcholine^24^. Cholinesterase inhibitors increase the overall amount of acetylcholine available. Thus, symptoms of overstimulation of the parasympathetic nervous system, such as increased hypermotility, hypersecretion, bradycardia, miosis, diarrhea, vomiting, nausea, and hypotension, may be present^24^. Drugs currently used for treating AD still, very commonly, cause such symptoms even in patients with mild AD. Adverse effects are more frequent and prominent when the disease progresses^25^. Considering this and the results of our study showing improved memory, cognitive functions, and learning abilities, the new BChEi used in our study could represent a viable alternative to the currently available AD treatments in humans, potentially causing less cholinergic side effects during treatment.

One limitation of our study is that we did not have a placebo group. Therefore, the owner’s ratings of the dogs’ behavior may have been subjective. We tried to minimize this with regular clinical check-ups, evaluating the dogs’ behavior always by the same veterinarian, including a questionnaire about the dog’s cognitive impairment that was filled by a family member or a friend not living in the same household, and with repeating the problem-solving tests. The placebo group would add much more value to the results of our study. However, it was difficult to find older dogs without kidney or liver damage or other systemic disease, and owners willing to participate in the test treatment with completely novel substance. Owners came for help, they were in great distress, and most dogs had to be excluded due to medical conditions that are normal in older dogs and could interfere with the treatment. In addition, the possible side effects and the effects of the novel drug on kidney and liver function were not yet known at the beginning of the study and it was therefore difficult to convince the owners to participate in the study with such novel compound blindly. With early recognition of cognitive dysfunction in older dogs by veterinarians and owners, and with preliminary data from the current study that the compound is safe, more dogs could be included in the future studies, which should be placebo-controlled.

Although prospective studies (especially randomized controlled trials) in which the outcome is unknown at the time of recruitment, such as ours, are less prone to selection bias, there were some points of potential bias during the study. These include: no placebo group, questionnaire and test were administered by a person who knew the dogs' status, although a second person who did not know the status scored the behavioural tests. With early recognition of cognitive dysfunction in older dogs by veterinarians and owners, more dogs could be included in future studies, which should be randomized and placebo-controlled. In addition, bias should be avoided as much as possible in every step of the study, including study design or data collection, as well as data analysis and publication. Therefore, careful planning of the study design and data analysis should be done at the beginning of the study. To avoid questionnaire bias, standardized protocols for data collection, including training of study personnel, should be implemented to minimize interobserver variability when multiple individuals are collecting and entering data. Blinding of study personnel to patient exposure and outcome status or, if this is not possible, measurement of outcome by investigators other than those who assessed exposure, should also be considered.

In conclusion, our results show that BChEi treatment improved cognitive function in dogs with the moderate cognitive decline with no side effects. Overall, owners experienced an enhanced quality of life for treated dogs and themselves. These results provide encouraging pre-clinical data to support further clinical studies that should include placebo-controlled, randomized groups of dogs with mild, moderate, and severe cognitive impairment. We have also briefly outlined that the disease is still underdiagnosed and that owners could not recognize CCD in its early stage. Early recognition of cognitive aging and particularly cognitive dysfunction in aged pet dogs is of great importance since it seems that the appropriate treatment needs to be started early to be most effective.

## Materials and methods

### Synthesis of the drug (Compound 1)

**Figure 4:**
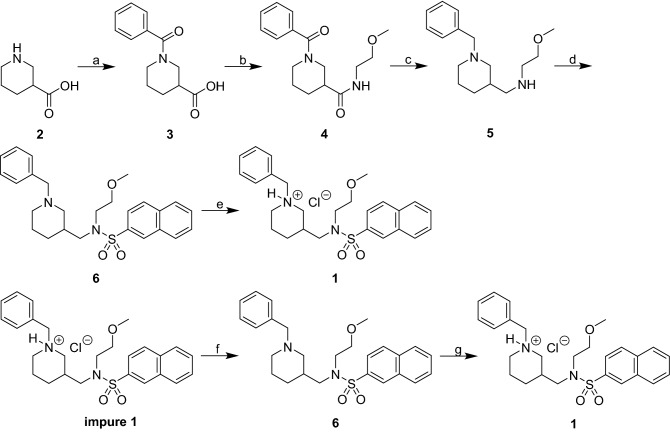
Synthesis of compound. Reagents and conditions: (**a**) (i) BzCl, K_2_CO_3_, THF/H2O, 0 °C to room temperature, 24 h, (ii) 6 M HCl_(aq)_ to pH 1–2, 0 °C, 2 h (93%); (**b**) H_2_N(CH_2_)_2_OMe, TBTU, Et_3_N, CH_2_Cl_2_, 0 °C to room temperature, 24 h (86%); (**c**) LiAlH4, dry THF, room temperature to reflux, under Ar_(g)_, 3.5 h (93%); (**d**) naphthalene-2-sulfonyl chloride, Et_3_N, CH_2_Cl_2_, 0 °C to room temperature, 24 h (95%); (**e**) 2 M HCl solution in Et_2_O, MeOH, 0 °C to room temperature, 24 h (69%); (**f**) (i) H_2_O, 2 M NaOH(aq) to pH 12–13, 0 °C, (ii) extraction into CH_2_Cl_2_, (iii) purification via column chromatography (78%); (**g**) 2 M HCl solution in Et_2_O, MeOH, 0 °C to room temperature, 24 h (79%).

The drug was synthesized from piperidine-3-carboxylic acid in a 64% overall yield using a modified previously described procedure^17,26^ (Fig. 4). To prevent quick elimination from the body, sustained-release tablets were used for experimental therapy. A detailed description of the compound synthesis and tablet preparation is provided in Supplement 1.

### Dog recruitment

Thirty-one dogs older than 10 years were screened for the study. Initial diagnosis was based on signalment, history, clinical features, physical and neurological examination, CADES questionnaire and two cognitive tests: food searching test (FST) and problem-solving test (PST). Blood work (CBC and clinical chemistry) and urinalysis were performed to rule out geriatric disorders that can mimic signs of cognitive impairment. After thorough examinations, the decision was made whether the dogs were included in the study. Dogs with systemic illnesses that could interfere with their cognitive function were excluded from the study. A flowchart of recruitment is presented in Fig. 1.

Only dogs aged 10 years or older were included in the study. The age of the dogs was determined based on the information in the Slovenian register, chip number and dogs’ passports. All dogs displayed at least mild cognitive impairment according to CADES score. Dogs were not treated with any other medications for cognitive impairment. However, dogs that were included in the control group were allowed to be fed with the commercial therapeutic diet for cognitive decline, if they were on it at least 8 weeks before entering the study and if they maintained the same dietary protocol for the duration of the study. Dogs in the treatment group that were on a diet meant to treat cognitive decline were required to be off the diet for at least 30 days before enrollment and during the duration of the study.

### Study design

Before starting the experimental therapy with their pets, each owner was required to sign an informed consent form. According to The Administration of The Republic of Slovenian for Food Safety, Veterinary and Plant Protection, no ethical permission was needed as the study was performed on clinical patients with the owners’ consents. In total, seventeen dogs showing signs of cognitive dysfunction were included in the study. Dogs were allocated to either a control group (n = 7) without treatment or the treatment group (n = 10). Questionnaire and cognitive tests were performed at the experimental facility every 3 months on all the dogs included in the study by two veterinarians who provided independent scores. One of the veterinarians was aware which dogs were treated and which were not, while the other veterinarian was not aware of the status of the dogs in regard to the treatment. After tests, scoring from both vets was compared independently and there was no difference in scoring. When the patient's condition deteriorated drastically and the owners decided to euthanize their dogs, the dog was examined by a veterinarian who was not involved in the study. The veterinarian who performed the clinical examination and tests in this study was not involved in any way in the decision to euthanize. Each treated dog received the BChEi twice daily for at least 6 months in concentration 10 mg/kg of the active substance.

### Physical and neurological examinations

Owners observed their dog’s health status daily. In case of any serious or abnormal observations, they contacted the participating veterinarian. Physical and excessive neurological examinations (behavior and consciousness, cranial nerves, gait and posture, postural reactions, spinal reflexes) were carried out by the veterinarian and included an assessment of all body systems. A full spectrum of neurobehavioral symptoms including apathy, anxiety, staring blankly, confusion or aimless walking, vocalization during the night, aggression, signs of compulsive and stereotyped behavior, etc., were obtained by questioning the pet owners. Dogs were monitored during examinations. Their responses to familiar and unfamiliar people and objects were observed. Exams were performed at the beginning and every three months for the duration of the study.

### Behavior examination

Changes in the behavior of all dogs were evaluated by the same veterinarian (MZP). Each check-up included observations of the dog and the collection of information provided by its owner. Questions focused on information about appetite, drinking, loss of perception (attempts to pass through narrow spaces or the wrong side of the door), disorientation, nigh-time waking, anxiety, excessive vocalization, house soiling, aggression, changes in activity, attention-seeking behavior, aimless behavior (stargazing, circling, stereotyped walking), panting, muscle tremors/shaking, memory loss, and any abnormal signs. To quantify the cognitive decline in dogs, we used a scoring system—the Canine Dementia Scale (CADES), adapted and modified from Osella et al. (2007), Salvin et al. (2010), and Madari et al. (2015)^5,19,27^. The questionnaire was filled out by the clinician and it was divided into four domains according to different ability areas: domain A for spatial orientation, domain B for social interactions, domain C for house soiling, and domain D for sleep–wake cycles (Table 1). After completion and scoring of the questionnaires, canine patients were classified into four stages (mild, moderate, severe, and extreme cognitive impairment). The questionnaire is provided as Supplement 2.

### Cognitive tests

Food search test (FST) and problem-solving test (PST) were performed on each dog included in the study. The design of these tests was based on previously published tests for CCD^21^. The cognitive tests were evaluated by the same veterinarian (MZP). Dogs were tested in the morning after at least 10 h of the fasting period. A thorough description of the tests, together with the explanation of the scoring system and individual scores, is provided in Supplement 3.

### Blood work and urinalysis

Complete hematology with cytology was performed to investigate the presence of anemia, abnormal white blood cell count, or abnormal platelet count. Serum biochemistry was performed to evaluate renal (urea, creatinine) and liver parameters (ALT, ALP, AST), and also included the measurement of serum glucose, serum cholesterol, serum albumin, globulin, sodium, and calcium. Urinalysis was also performed. In dogs included in the treatment group, the blood work and urinalysis were performed every two weeks to ensure the drug did not affect kidney and liver function. For these additional analyses, samples were collected by another veterinarian (DP) and at different facilities at Veterinary Faculty. This was done to prevent the treated dogs from becoming familiar with the test site and personnel when the cognitive tests were performed and evaluated.

### Statistical analysis

Results are presented as mean ± SD. Age between groups was compared by one-way ANOVA. CADES score and results from behavioral tests were analyzed by repeated measures ANOVA with treatment as the independent variable and clinical test (before the treatment, 3 months, and 6 months) as within factor. Change in CADES score and behavioral tests score during the study was tested separately in the treated and untreated groups by one-way ANOVA with the time of clinical testing as an independent variable. Differences were considered statistically significant with *p* < 0.05.

### Ethics approval and consent to participate

According to The Administration of The Republic of Slovenian for Food Safety, Veterinary and Plant Protection, no ethical permission was needed as the study was performed on clinical patients with the owners’ consents.

## Supplementary Information

Supplementary Information.

## Data Availability

All data is provided in the manuscript or supplementary material.

## Abbreviations

AD: Alzheimer’s disease
BChE: Butyrylcholinesterase
BChEi: Butyrylcholinesterase inhibitor
CCD: Canine cognitive dysfunction
CADES: Canine Dementia Scale
CDS: Cognitive dysfunction syndrome
FST: Food search test
PST: Problem-solving test
