# Supplementary material for: Treatment of canine cognitive dysfunction with novel butyrylcholinesterase inhibitor
Source: Sci Rep. 2021 Sep 13;11:18098. doi: 10.1038/s41598-021-97404-2 (PMC8438013; doi:10.1038/s41598-021-97404-2)
Supplement: Supplementary file 1 — Supplementary Information. [file 41598_2021_97404_MOESM1_ESM.pdf]

Treatment of canine cognitive dysfunction with novel butyrylcholinesterase inhibitor

Maja Zakošek Pipan*, Sonja Prpar Mihevc†, Malan Štrbenc†, Urban Košak‡, Ilija German Ilić‡, Jurij Trontelj‡, Simon Žakelj‡, Stanislav Gobec‡, Darja Pavlin§, Gregor Majdič†

*Veterinary Faculty, Clinic for Reproduction and Large Animals, University of Ljubljana, Gerbičeva 60, Ljubljana, Slovenia; †Veterinary Faculty, Institute for Preclinical Sciences, University of Ljubljana, Gerbičeva 60, Ljubljana, Slovenia; ‡Faculty of Pharmacy, University of Ljubljana, Aškerčeva cesta 7, Ljubljana, Slovenia; §Veterinary Faculty, Clinic for Small Animals, University of Ljubljana, Gerbičeva 60, Ljubljana, Slovenia

Running title: Treatment of canine dementia with BCHE inhibitor

ORIGINAL RESEARCH article

* Corresponding author:
Prof. dr. Gregor Majdič
Institute for Preclinical Sciences, Veterinary Faculty, University of Ljubljana
Gerbičeva 60
1000 Ljubljana
Slovenia
Tel.: +386 14 779 210
e-mail: gregor.majdic@vf.uni-lj.si

SUPPLEMENT 1: Preparation of drug

1.1 Synthesis of 1-benzoylpiperidine-3-carboxylic acid (3)

To a 4-L round-bottomed flask equipped with a stirring bar, piperidine-3-carboxylic acid (**2**) (100 g, 0.774 mol, 1.0 equiv) was added. THF (600 mL) was added and the resulting suspension was stirred. H_2O (800 mL) was added, and after all compound **2** dissolved, the solution was cooled to 0 °C. K_2CO_3 (536 g, 3.878 mol, 5.0 equiv) were added portion-wise. A solution of benzoyl chloride (90 mL, 0.774 mol, 1.0 equiv) in THF (200 mL) was then added drop-wise. The reaction mixture was allowed to warm to room temperature, stirred for 24 hours, transferred into a 2-L separating funnel and washed with EtOAc (3 × 1 L). The aqueous phase was transferred into a 5-L beaker equipped with a stirring bar, stirred, cooled to 0 °C, and adjusted to pH 1-2 with 6 M aqueous HCl solution. A white solid precipitated and the suspension was stirred at 0 °C for 2 hours. The white precipitate was then collected in a Büchner funnel under suction filtration, washed with H_2O (3 × 500 mL) and dried in a drying oven at 80 °C to constant mass to produce 169 g of **3** (93 % yield). This product was used in the next step without further purification. Compound **3** was characterized using the materials and methods described previously (Košak et al., 2014). The characterization data for compound **3** was in accordance with the previously reported characterization data (Košak et al., 2014).

1.2. Synthesis of 1-benzoyl-N-(2-methoxyethyl)piperidine-3-carboxamide (4)

To a 4-L round-bottomed flask equipped with a stirring bar, **3** (169 g, 0.725 mol, 1.0 equiv) was added followed by CH_2Cl_2 (3 L). The resulting suspension was stirred and cooled to 0 °C. Et_3N (202 mL, 1.449 mol, 2.0 equiv) was added drop-wise. After all the solid dissolved, *O*-(benzotriazol-1-yl)-*N*,*N*,*N'*,*N'*-tetramethyluronium tetrafluoroborate (TBTU) (233 g, 0.725

mol, 1.0 equiv) was added in two equal portions. After 1 hour, 2-methoxyethylamine (125 mL, 1.449 mol, 2.0 equiv) was added drop-wise via a dropping funnel. The reaction mixture was allowed to warm to room temperature, stirred for 24 h and then divided up into 3 portions of approximately 1 L. Every portion was transferred into a 2-L separating funnel, washed with H_2O (2 × 1 L), 0.5 M aqueous HCl solution (2 × 1 L) followed by saturated aqueous $NaHCO_3$ solution (2 × 1 L), and dried over anhydrous Na_2SO_4. All dried organic phases were pooled and evaporated, to produce 186 g of **4** as a colourless oil (86 % yield). This product was used in the next step without further purification. Compound **4** was characterized using the materials and methods described previously (Košak et al., 2014). The characterization data for compound **4** was in accordance with the previously reported characterization data (Košak et al., 2014.

1.3 Synthesis of N-((1-benzylpiperidin-3-yl)methyl)-2-methoxyethan-1-amine (5)

To a 1-L tree-neck round-bottomed flask equipped with a stirring bar and a reflux condenser, $LiAlH_4$ (14.200 g, 0.374 mol, 3.5 equiv) was added under an argon atmosphere. Dry THF (ca. 450 mL) was added with a double-tipped needle. A solution of **4** (31 g, 0.107 mol, 1.0 equiv) in dry THF (ca. 150 mL) was added with a double-tipped needle, and the reaction mixture was refluxed for 3.5 hours. The mixture was then cooled to 0 °C and the excess hydride was decomposed by drop-wise addition of H_2O (14.2 mL) followed by 15% aqueous NaOH solution (14.2 mL) and then H_2O (42.6 mL). The suspension was allowed to warm to room temperature, stirred for 12 h then filtered under suction. The white precipitate was washed thoroughly with THF (5 × 200 mL). This reaction was performed in the same way 5 more times to use up all of compound **4**. Filtrates of all 6 reactions were pooled together and evaporated to produce 156 g of **5** as a slightly golden liquid (93 % yield). This product was used in the next step without further purification. Compound **5** was characterized using the materials and

methods described previously (Košak et al., 2014). The characterization data for compound **5** was in accordance with the previously reported characterization data (Košak et al., 2014).

1.4. Synthesis of N-((1-benzylpiperidin-3-yl)methyl)-N-(2-methoxyethyl)naphthalene-2-sulfonamide (6)

To a 2-L round-bottomed flask containing **5** (156 g, 0.595 mol, 1.0 equiv), CH_2Cl_2 (1.5 L) was added. A stirring bar was added to the resulting solution, which was then stirred and cooled to 0 °C. Et_3N (83 mL, 0.595 mol, 1.0 equiv) was added drop-wise. After 30 minutes, naphthalene-2-sulfonyl chloride (135 g, 0.595 mol, 1.0 equiv.) was added portion-wise. The reaction mixture was allowed to warm up to room temperature, stirred for 24 hours and divided up into 2 portions of approximately 1 L. Every portion was transferred into a 2-L separating funnel, washed with H_2O (1 L), followed by 1 M aqueous NaOH solution (1 L), and dried over anhydrous Na_2SO_4. Both dried organic phases were pooled together and evaporated, to produce 256 g of **6** as a slightly golden oil (95 % yield). This product was used in the next step without further purification.

1.5 Synthesis of N-((1-benzylpiperidin-3-yl)methyl)-N-(2-methoxyethyl)naphthalene-2-sulfonamide hydrochloride (1)

To a 2-L round-bottomed flask containing **6** (128 g, 0.282 mol, 1.0 equiv), MeOH (640 mL) was added. A stirring bar was added to the resulting solution, which was then stirred, agitated with a stream of argon for 30 min, and cooled to 0 °C. 2 M HCl solution in Et_2O (156 mL, 0.310 mol, 1.1 equiv) was added with a double-tipped needle. The reaction mixture was allowed to warm up to room temperature, stirred for 24 hours and evaporated. MeOH (240 mL) was added to the residue, followed by a stirring bar. The solution was stirred and Et_2O (1.5 L)

was added slowly. A white solid precipitated and the suspension was stirred at room temperature for 3 hours. The white precipitate was then collected in a Büchner funnel under suction filtration and washed with Et_2O (2 × 500 mL). This reaction was performed in the same way with the rest of **6** (128 g). The white solid from both batches were pooled together and dried in a desiccator *in vacuo* at room temperature in the presence of crushed NaOH to constant mass to produce 192 g of **1** (69 % yield). Compound **1** was characterized using the materials and methods described previously (Košak et al., 2016). The characterization data for compound **1** was in accordance with the previously reported characterization data (Košak et al., 2016). The mother liquids from both crystallizations were pooled together and evaporated to produce 84 g of impure **1** as a slightly golden oil.

1.6. Purification of impure compound 1

To a 2-L round-bottomed flask containing impure **1** (84 g), H_2O (840 mL) was added. A stirring bar was added to the mixture which was then stirred, cooled to 0 °C, and adjusted to pH 12-13 with 2 M aqueous NaOH solution. The mixture was transferred into a 2-L separating funnel and extracted with CH_2Cl_2 (2 × 1 L). The combined organic phases were washed with 1 M aqueous NaOH solution, dried over anhydrous Na_2SO_4, and evaporated. The residue was purified by flash column chromatography using CH_2Cl_2-MeOH (30:1) then CH_2Cl_2-MeOH (10:1) as the eluent to produce 57 g of **6** as a slightly golden oil. Compound **6** was characterized using the materials and methods described previously (Košak et al., 2016). The characterization data for compound **6** was in accordance with the previously reported characterization data (Košak et al., 2016).

1.7. Synthesis of N-((1-benzylpiperidin-3-yl)methyl)-N-(2-methoxyethyl)naphthalene-2-sulfonamide hydrochloride (1)

To a 1-L round-bottomed flask containing **6** (57 g, 0.126 mol, 1.0 equiv), MeOH (300 mL) was added. A stirring bar was added to the resulting solution which was then stirred and agitated with a stream of argon for 30 min, and cooled to 0 °C. 2 M HCl solution in Et_2O (69 mL, 0.139 mol, 1.1 equiv) was added with a double-tipped needle. The reaction mixture was allowed to warm up to room temperature, stirred for 24 hours and evaporated. Et_2O (500 mL) was added to the oily residue, and the flask was placed in an ultrasonic bath for 30 min. During this time, the oily residue transformed into a white solid. A stirring bar was added and the suspension was stirred for 12 hours at room temperature. The white precipitate was then collected in a Büchner funnel under suction filtration, washed with Et_2O (2 × 500 mL) and dried in a desiccator *in vacuo* at room temperature in the presence of crushed NaOH to constant mass to produce 49 g of **1** (79 % yield). Compound **1** was characterized using the materials and methods described previously (Košak et al., 2016). The characterization data for compound **1** was in accordance with the previously reported characterization data (Košak et al., 2016). This batch of **1** was pooled together with the previously synthesized batch of **1** to produce a total of 241 g of **1** (64 % overall yield from **2**).

1.8. Preparation of tablets

Tablets were prepared as follows: Tableting mixture composition (w/w): 63.0% of active ingredient, 35.0% of hypromellose (Methocel® K100M, Dow Chemical Company, MI, USA), 1.0% sodium lauryl sulphate (Sigma-Aldrich) and 1.0% magnesium stearate (Peter Greven). All materials were sieved through 400 µm sieve. Active ingredient, hypromellose and sodium

lauryl sulphate were weighted into a glass container and mixed manually for 15 minutes, then magnesium stearate was added and mixed for 3 minutes.

Single-punch tableting press (Kilian SP300, Kilian GmbH, Cologne, Germany) with 8.0 mm diameter biconvex round punch was used to compress 159 mg tablets (containing assay of 100 mg of active ingredient) at compression force of 5.5 – 6.0 kN (corresponding to 110–125 N of tablet hardness).

The in vitro dissolution profiles were obtained with a USP II dissolution apparatus (VK7000, VanKel, USA) in two dissolution media: 0.01M HCl and phosphate buffer pH 6.8 with 0.25% SDS. The profiles were generated using 900 mL medium with 100 rpm at 37 °C with 10 sampling time points over 24 hours. The sample concentrations were analyzed by an Agilent 1100 HPLC system (degasser, binary pump, thermostated well plate sampler, column thermostat, diode-array detector). A Phenomenex Kinetex 2.6 µm C18 50×4.6 mm column thermostated to 40 °C was used for the analysis with an isocratic mobile phase A : B = 63% : 37% (A: 0.5% ammonium phosphate buffer pH = 3.0 and B: Acetonitrile) flowing at 2.0 mL/min. The sample injection volume was 10 µL and the UV detection wavelength was 231 nm.

1. Košak U, Brus B, Gobec S. Straightforward synthesis of orthogonally protected piperidin-3-ylmethanamine and piperidin-4-ylmethanamine derivatives. Tetrahedron Lett. 2014; 55(12):2037–2039.
2. Košak U, Brus B, Knez D, et al. Development of an *in-vivo* active reversible butyrylcholinesterase inhibitor. Sci Rep. 2016;6:39495.

SUPPLEMENT 2: CADES questionnaire and basic information about patients included in the study

Table 1: Assessment of cognitive decline based on the modified CADES scale system adapted by Madari et al., 2015 [22].

ABNORMAL BEHAVIOR					
	Never observed	At least 1x/month	At least 1x/week	Several times/week	At least 1x/day
	0 points	2 points	3 points	4 points	5 points
Domain A: Spatial orientation – SCORE (0-25)					
Disorientation in a familiar environment (inside or outside, staring at the floor, table, etc.)					
Failure to recognize familiar people and animals (inside or outside)					
Abnormally respond to familiar objects					
Aimlessly wandering (walking around in circles)					
Reduced ability to do tasks previously learned					
Domain B: Social interaction – SCORE (0-25)					
Changes in interaction a man/dog/other dog (playing, petting, welcoming, increased reassurance seeking)					
Changes in individual behavior of dog (exploration behavior, play, performance)					
Response to commands and ability to learn new task					
Irritable/increased anxiety					
Changed patterns of aggression					
Domain C: House soiling					

Elimination at random locations at home					
In its sleeping area					
Changes of signalization for elimination activity					
Elimination indoors after a recent walk outside					
At uncommon locations					
Domain D: Sleep-wake cycles					
	0 points	4 points	6 points	8 points	10 points
Abnormal night time activity (wandering, localization, restless)					
Switch from insomnia to hypersomnia					

Total score (A + B + C + D) = 0 – 95

Clinical stage:

Score 0–7 = Normal ageing

Score 8–23 = Mild cognitive impairment

Score 24–44 Moderate cognitive impairment

Score 45–69 = Severe cognitive impairment

Score 70–95 = Extreme cognitive impairment

Table 2: Breed and age of patients included in the study and the CADES scores in untreated and treated patients

**Untreated patients**			**CADES score**		
**Patient Number**	**Breed**	**Age (Years)**	**At the beginning**	**After three months**	**After six months**
1	Pug	13	65	85	euthanasia
2	American Stafford	14.5	37	52	81
3	Cross breed	14	24	54	euthanasia
4	Cross breed	14	32	55	euthanasia
5	Maltese Dog	17	33	64	euthanasia
6	Cross breed	16	62	78	80
7	Maltese Dog	15	53	60	euthanasia
**Treated patients**			**CADES score**		
**Patient Number**	**Breed**	**Age**	**At the beginning**	**After three months**	**After six months**
1	Bull Terrier	13.5	44	38	14
2	Cross breed	13.5	29	22	16
3	Cross breed	13	36	20	16
4	Standard Schnauzer	13	19	8	8
5	Dachshund	14	27	10	10
6	Tibetan Terrier	16	30	10	12
7	Tibetan Terrier	16	24	12	12
8	Maltese Dog	13	54*	exclusion	exclusion
9	Dachshund	16	62*	exclusion	exclusion
10	Pug	13.5	65*	exclusion	exclusion

Legend: * treatment was stopped immediately because of the drug side effect

SUPPLEMENT 3: Behavioral tests

3.1. Food searching test (FST)

This test was based on testing the dog's ability to find hidden food. The dog was seated in the middle of the room while being on the leash. The veterinarian was positioned in front of the dog (60 cm away) and showed the dog a piece of its favorite treat. While maintaining visual contact with the dog and still communicating with the dog, showing the food, the veterinarian than moved backward and placed the food in the corner of the room. The veterinarian than stared at the food and pointed to it with his hand for 2-3 s to increase the dog's visual processing. The owner was then asked to leave the room with the dog and wait outside for 15 s. After returning to the room, the dog was placed into the center of the room unleashed and allowed to freely explore the room for 1 minute. No verbal or other clues were allowed. The procedure was repeated twice. In each run the test was scored as follows: the dog goes directly to the food (1 point), the dog searches for the food and finds it within 1 minute (2 points); the dog searches for the food but does not find it within 1 minute (3 points); the dog does not make any attempt to search for the food (4 points). The FST total score was the average of both runs.

3.2. Problem solving test (PST)

In this test, the ability of the dog to obtain the food by manipulating an object was assessed. The owner showed the dog its favorite food and the dog was allowed to sniff and lick it. After that, the food was placed on the floor in front of the dog and covered with a transparent plastic box that had been turned upside down. The dog was given 2 minutes to find a way to remove the box and get the food. The test was performed twice and scored as follows: the dog obtained the food within 2 minutes (1 point), the dog tried to obtain the food but did not obtain it within 2 minutes (2 points), the dog sniffed the box but did not try to get the food (3 points), the dog

did not make any attempt to get the food (4 points). The PST total score was the average of both runs. The sum of FST and PST test scores was used for the scoring and the statistical analysis.

3.3. Results of behavioral tests

The test score results for food searching and problem solving tests in untreated and treated dogs are summarized in the Table 3.

Table 3: Problem solving test scores (sum of FST and PST test) in dogs with cognitive impairment (* dogs excluded from the study due to gastrointestinal problems)

**Untreated group**	**Test results**		
**Patient Number**	**At the beginning**	**After three months**	**After six months**
1	8	8	euthanasia
2	4	8	8
3	2	8	euthanasia
4	8	8	euthanasia
5	3	5	euthanasia
6	6	8	8
7	8	8	euthanasia
**Treated group**	**Test results**		
**Patient Number**	**At the beginning**	**After three months**	**After six months**
1	4	4	2
2	5	4	3
3	6	2	2

4	4	2	2
5	7	4	4
6	5	4	4
7	4	2	2
8	4*	excluded	excluded
9	6*	excluded	excluded
10	8*	excluded	excluded
